# Compressive confocal microscopy imaging at the single-photon level with ultra-low sampling ratios

**DOI:** 10.1038/s44172-024-00236-x

**Published:** 2024-06-25

**Authors:** Shuai Liu, Bin Chen, Wenzhen Zou, Hao Sha, Xiaochen Feng, Sanyang Han, Xiu Li, Xuri Yao, Jian Zhang, Yongbing Zhang

**Affiliations:** 1 Tsinghua Shenzhen International Graduate School, Tsinghua University, Shenzhen, 518055 China; 2 School of Electronic and Computer Engineering, Peking University Shenzhen Graduate School, Shenzhen, Guangdong 518055 China; 3 School of Computer Science and Technology, Harbin Institute of Technology (Shenzhen), Shenzhen, Guangdong 518055 China; 4 Center for Quantum Technology Research, School of Physics, Beijing Institute of Technology, Beijing, 100081 China

**Keywords:** Imaging and sensing, Fluorescence imaging

## Abstract

Laser-scanning confocal microscopy serves as a critical instrument for microscopic research in biology. However, it suffers from low imaging speed and high phototoxicity. Here we build a novel deep compressive confocal microscope, which employs a digital micromirror device as a coding mask for single-pixel imaging and a pinhole for confocal microscopic imaging respectively. Combined with a deep learning reconstruction algorithm, our system is able to achieve high-quality confocal microscopic imaging with low phototoxicity. Our imaging experiments with fluorescent microspheres demonstrate its capability of achieving single-pixel confocal imaging with a sampling ratio of only approximately 0.03% in specific sparse scenarios. Moreover, the deep compressive confocal microscope allows single-pixel imaging at the single-photon level, thus reducing the excitation light power requirement for confocal imaging and suppressing the phototoxicity. We believe that our system has great potential for long-duration and high-speed microscopic imaging of living cells.

## Abstract (web summary)

Shuai Liu, Bin Chen and colleagues improve imaging speed and reduced phototoxicity in confocal microimaging by building a deep compressive confocal microscope. Digital micromirror acts as a coding mask for deep learning-based reconstruction algorithms.

## Introduction

Fluorescence microscopy is a pivotal tool for monitoring cell physiology and studying various biological problems^1^. A major issue with fluorescence microscopy is the interference of out-of-focus light, which results in the blurring of details and reduction of contrast^2,3^. This problem is commonly resolved by optical sectioning techniques, which eliminate the out-of-focus light from the in-focus imaging scene^4–6^. Laser-scanning confocal microscopy is the most widely used observation instrument for fluorescence imaging^7,8^. However, the imaging speed of laser-scanning confocal microscopy is slow since it adopts the point-by-point imaging method and requires a higher-power laser to enhance the signal-to-noise ratio, which causes bleaching of fluorescent markers and photodamage to the sample^9^. To solve these problems, researchers have proposed light sheet microscopy^10,11^ and spinning disk confocal microscopy^12,13^. Nevertheless, light sheet microscopy requires special preparation and fixation processes. Spinning disk confocal microscopy is limited in its flexibility and suffers from fluorescence crosstalk between pinholes. Another more flexible confocal microscope is the programmable array microscope (PAM)^14,15^, which uses a digital micromirror device (DMD) as a pinhole for multi-point parallel scanning and has programmable properties. However, the illumination beam of the PAM needs to be extended to cover the entire surface of the DMD, thus reducing the excitation efficiency. Hence, it requires extending the measurement time or using a higher-power laser illumination source. Moreover, all of the light sheet microscopy, spinning disk confocal microscopy and PAM require highly sensitive detector arrays, such as the complementary metal-oxide-emitter semiconductor (sCMOS) or the electron-multiplying charge-coupled device. Compared with single-pixel detectors, these detector arrays have a narrow spectral response range, a low temporal resolution, and a high deployment cost.

Single-pixel imaging (SPI)^16,17^ based on compressive sensing^18–20^ is an ideal imaging method to replace the detector array imaging in some scenarios due to its characteristics such as high-throughput measurement, under-sampling, and higher time resolution. Hence, SPI has found numerous applications in compressive radar^21^, X-ray imaging^22–24^, terahertz imaging^25–27^, infrared imaging^28^ and biomicroscopic imaging^29–32^. However, SPI still has the problem of low imaging speed, which results from the limited refresh rate of the spatial light modulator and multiple single-pixel sampling. To overcome this obstacle, researchers tried to replace the spatial light modulators with faster modulation schemes, such as a matrix of light-emitting diodes^33^ or a high-speed rotating cyclic Hadamard mask^34^. However, these methods cannot flexibly change either the light source or the modulation mode. Others exploited the information related to the dynamic scene with a static mask to reconstruct a series of time-varying images of the scene^31,35^. Although this approach improves the imaging speed, it requires high-speed movement of the samples or a repetitive dynamic scene. On the other hand, deep learning algorithms can reduce the required measurements for reconstruction^36^ and can be applied to any kind of SPI system. However, the current deep learning-based SPI typically utilizes simulated or unpaired datasets to train the network^37,38^, which may result in unreliable reconstructed images in the actual experimental setup. In summary, the low imaging speed and unsatisfactory imaging quality are currently the main limitations hindering the development and practical applications of SPI.

In this study, we present a deep compressive confocal microscope (DCCM) in conjunction with a deep learning reconstruction algorithm (DCCM-Net) to achieve confocal microscopic imaging at the single-photon level with ultra-low sampling ratios. The DCCM-Net is a novel and powerful deep unrolling reconstruction algorithm under the framework of the interpretable proximal gradient descent (PGD) optimization model^39,40^. The DCCM can provide single-pixel compressed data and confocal images in pairs for DCCM-Net training. The DCCM-Net trained by real sampled data is able to achieve high-quality confocal microscopic imaging with low phototoxicity. To further improve the imaging speed, we introduce an innovative high-speed zoom imaging mode, comprised of a neighborhood merging compressed sampling and a neighborhood unmerging reconstruction. Under the high-speed zoom imaging mode, the DCCM allows confocal imaging of the fluorescent microsphere at a sampling ratio of 0.03% as well as Nucleus and F-actin at a ratio of 0.4%. For an image with 128 × 128 pixels, our DCCM can achieve imaging rates of up to 1500 fps and 24.58 megapixels per second based on the DMD with the refresh rate of 15,000 Hz. Notably, the imaging speed could be further increased to 3200 fps if we use the DMD with the highest refresh rate (32 kHz) currently available^41^. Furthermore, we employ a high-throughput aggregation collection to achieve weak signal detection at the single-photon level using a highly sensitive photon counter detector. In experiments, our compressive imaging can be accomplished with an average of no more than 0.4 photons per pixel in a single-pattern measurement. Consequently, the phototoxicity can be reduced by our DCCM, since it does not require high excitation light power. In total, this study provides a fast confocal microscopy imaging method based on the principle of SPI and our work has the potential to be applied in 3D microscopic imaging of thick fluorescent samples and living cells.

## Results

### Principle of DCCM

**Fig. 1:**
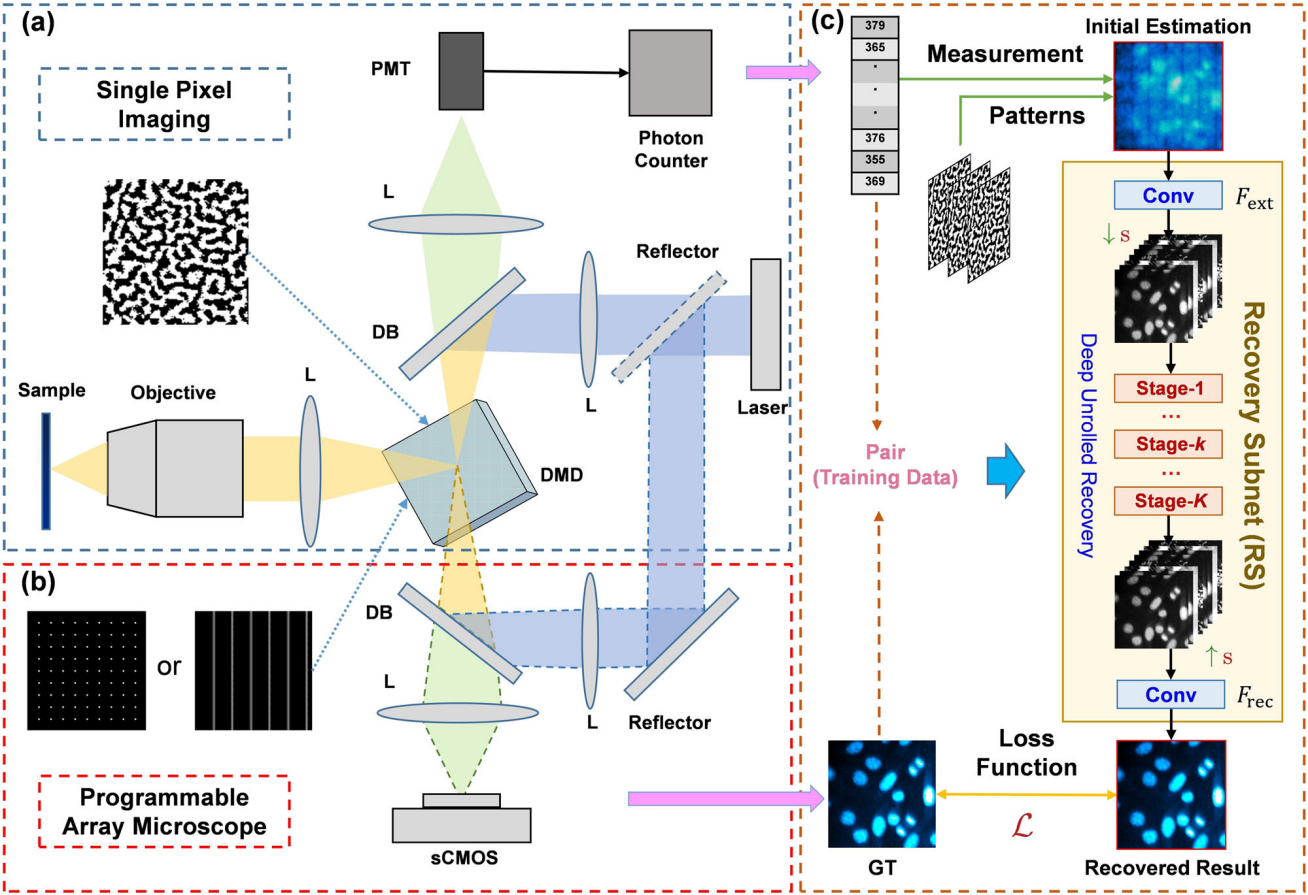
Workflow overview of the deep compressive confocal microscope. Our system consists of three parts **(a)**, **(b)**, and **(c)**. **a** Schematic of single-pixel imaging setup. A sequence of modulation patterns is adaptively learned for compressive single-pixel imaging sampling. **b** Schematic of programmable array microscope. Multi-point or multi-line patterns are utilized to implement the pinhole function to obtain the confocal images, which act as the ground truth for the following network training. **c** The overall structure of our deep learning reconstruction algorithm (DCCM-Net). The dataset for training comes from the modulation patterns for single-pixel imaging as well as the registered measurement and image data pairs captured by our single-pixel imaging and programmable array microscope systems in (**a**) and (**b**), respectively. In the actual application, only **a** and **c** are employed and activated to achieve high-speed and low-phototoxicity confocal microscopic imaging. L lens, DB dichroic beamsplitter, PMT photomultiplier tube, sCMOS scientific complementary metal-oxide-semiconductor, GT ground truth, s scaling factor, ${F}_{{{{{{\rm{ext}}}}}}}$ extraction module, ${F}_{{{{{{\rm{rec}}}}}}}$ recovery module, Conv convolution.

An illustration of our experimental setup is shown in Fig. 1. The system consists of a hardware device (DCCM) and a deep learning-based reconstruction algorithm (DCCM-Net). DCCM is a combination of SPI and PAM. Exploiting the advantages of the symmetrical reflective properties of DMD, we symmetrically place the SPI and PAM according to the reflective angle of DMD. In this way, the DMD can serve as a coding mask for SPI as well as a pinhole for confocal microscopic imaging. Therefore, the DCCM can capture single-pixel compressed data and confocal image data in pairs for network training. Our network is trained on the pairwise data collected by the DCCM system rather than the simulated data, enabling the model to consider the influence of noise distribution in real scenarios. Moreover, our experiments demonstrate that the DCCM-Net trained on real sampled data outperforms that trained on simulated data. (refer to Supplementary Fig. S1 for details).

### Experimental setup

Figure 1a schematically illustrates the experimental setup of SPI. The power tunable laser, operating at 405 nm or 488 nm, is used as the light source. The laser beam is reflected by a dichroic beamsplitter to the DMD (V7001 DLP7000&DLPC410), which modulates the laser and then illuminates the sample through an objective (Nikon 20×/0.75NA). The sample is selectively excited and emits fluorescence at the focal plane of the objective in accordance with the DMD modulation pattern. Note that the modulation pattern is a trainable component, which is adaptively learned from the sample (see “Methods” section). Subsequently, the fluorescence returns to the DMD along the excitation path and is focused onto a photon-counting photomultiplier tube (PMT, H10682-210 Hamamatsu) module, allowing weak signal detection. We use the PAM imaging, as shown in Fig. 1b, to acquire the confocal image, which is utilized as the ground truth for the training of DCCM-Net. To obtain confocal images with high quality, we turn the laser power to the maximum and set a longer exposure time for the sCOMS when the PAM module is activated. The sample is selectively excited according to the multi-point or multi-line pinhole pattern displayed by the DMD. The DMD only reflects the fluorescence in the white regions of the modulation patterns in Fig. 1b, and then the reflected fluorescence is recorded by the sCMOS (ORCA-Flash 4.0LT+ Hamamatsu) after a DB. Here, the DMD is used as a confocal pinhole because it is in the back focal plane of the objective lens. More details can be found in “Methods” section.

Figure 1c provides the architecture of the DCCM-Net for the image reconstruction. The DCCM-Net is based on the deep unfolding architecture^40^, which can efficiently estimate the confocal image from the corresponding single-pixel compressed measurement obtained by the SPI. As Fig. 1c exhibits, DCCM-Net takes the initial image estimation from the measurement and the sampling patterns as inputs and generates the non-linearly recovered result through a fast-forward pass. It contains several cascaded deep-stage modules sandwiched by two convolution layers. The stage modules share the same structure and are constructed by mapping the original hand-crafted optimization steps of PGD into convolutional neural network components, with each PGD iteration corresponding to a stage module. The details of the recovery subnet (RS) can be found in Supplementary Fig. S2. During training, the model parameters are jointly end-to-end iteratively updated on our collected paired and registered data from the DCCM system. Each data pair is augmented by randomly selecting some sets of compressed measurement elements and their corresponding sampling patterns. This strategy enables our method to handle the CS imaging tasks of arbitrary sampling ratios by using a single network model trained once. The details regarding the design of our DCCM-Net are provided in “Methods” section.

The DCCM can provide the DCCM-Net with a large number of training datasets from various samples or environments. This can improve the generalization and robustness of our network. Meanwhile, the DCCM-Net can also provide DCCM with optimal modulation patterns based on the dataset characteristics of different scenarios. This can improve the signal acquisition capability of the DCCM and realize confocal imaging with lower sampling ratios (see Supplementary Fig. S3 for details). Therefore, the DCCM and DCCM-Net can achieve positive feedback iteration.

### Performance of DCCM on cell samples

**Fig. 2:**
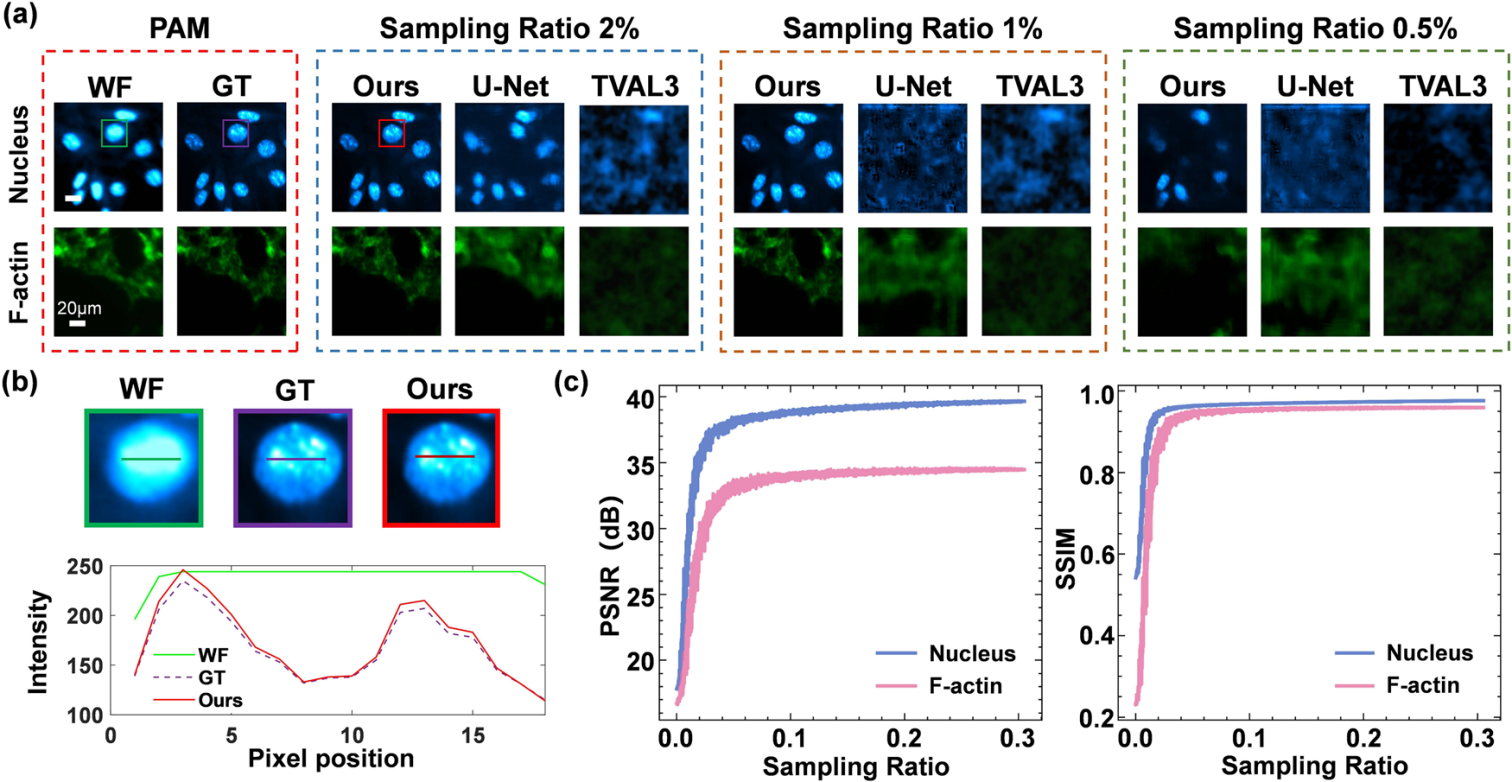
Experimental comparisons of various imaging methods at different sampling ratios. **a** Experiment results for Nucleus and F-actin. Each row within the group represents the reconstructed results of the same object by different methods, while each column depicts the results of different objects reconstructed by the same method. The pixel number is $N=128\times 128$, and the corresponding scale bars are 20 $\mu m$. **b** The enlarged views of the boxed areas in (**a**) and the intensity profile along the corresponding line trace. **c** The curves of our peak signal-to-noise ratio (PSNR) and structural similarity index measure (SSIM) scores with different sampling ratios. PAM programmable array microscope, WF wide field, GT ground truth.

To verify the performance of the DCCM, we perform experimental imaging of Nucleus and F-actin. Figure 2a displays the reconstructed results of the Nucleus and F-actin using three different reconstruction algorithms at sampling ratios of 0.5%, 1%, and 2%. The sampling ratio is calculated by dividing the number of sample patterns by the total number (16,384) of pixels of the image. The size of all reconstructed images shown in this study is 128 × 128, and the results of reconstruction at more sampling ratios are provided in Supplementary Fig. S4. We observe that the DCCM-Net has higher reconstruction quality than other conventional algorithms including U-Net^38^ and TVAL3^42^ at low sampling ratios. It provides an almost lossless reconstruction at a sampling ratio of 1% and achieves a higher resolution compared with wide-field imaging. Here, the wide-field images are obtained by the sCMOS with the DMD displaying an all-“on” pattern. From the DCCM-Net reconstruction results, we can clearly observe the nuclear speckles^43^ and more F-actin details. Figure 2b shows the enlarged views of the boxed areas in Fig. 2a and intensity profiles along the lines trace. The nuclear speckles can be clearly seen in the confocal image, acting as ground truth (GT) for comparison, directly obtained from PAM, while the wide-field image has a lower resolution. The red solid line (our network output) almost coincides with the purple dashed one (GT), which proves the reliability of the reconstruction by our method.

Compared with other deep learning-based reconstruction algorithms, our DCCM-Net is independent of the sampling ratios and thus can be applied to any SPI measurements without retraining the model again (see Supplementary Fig. S5 for details). For the reconstruction of the U-Net in Fig. 2, we need to train it three times at different sampling ratios of 0.5%, 1%, and 2%, while our network only needs to be trained once with the same amount of data required. In Fig. 2c, we demonstrate the results of Peak signal-to-noise ratio (PSNR) and structural similarity index measure (SSIM) scores under various sampling ratios. It is evident that the image reconstruction quality improves as the sampling ratio increases and reaches its peak when the sampling ratio exceeds approximately 2%. In addition, the reconstruction quality of F-actin images is lower compared to Nucleus probably due to the influence of complex morphological structures.

### The high-speed zoom imaging mode of DCCM

To further improve the imaging speed of DCCM, we introduce a high-speed zoom imaging mode, which consists of a neighborhood merging sampling and the corresponding neighborhood unmerging reconstruction. The neighborhood merging sampling is implemented by merging neighbor pixels in the modulation patterns, which scales the resolution of the modulation pattern while keeping the same field of view. For example, the 128 × 128 modulation pattern becomes 32 × 32 after the 4 × 4 pixels merging operation. Although this approach reduces the number of measurements required, it also leads to a lower resolution of the reconstructed image. To preserve the original imaging resolution of 128 × 128, we replace the merging sampling patterns with the neighborhood unmerging patterns as the input of DCCM-Net. The unmerging patterns are spatially upscaled sampling patterns of size 128 × 128 from the merged 32 × 32 ones used in SPI sampling by the nearest interpolation. Thus, DCCM enables confocal image reconstruction at ultra-low sampling ratios in zoom imaging mode.

**Fig. 3:**
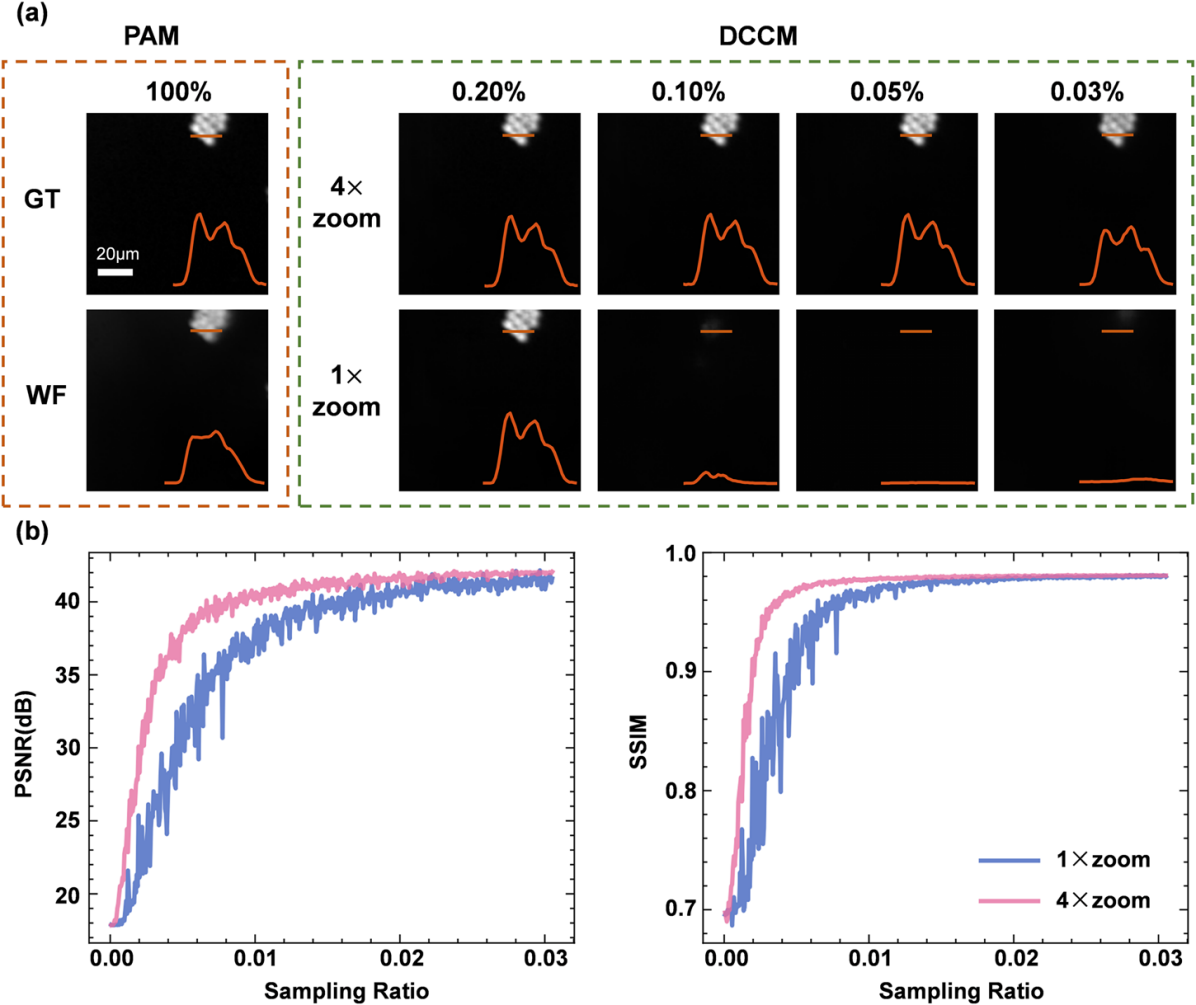
The high-speed zoom imaging of fluorescent microsphere. **a** Experimental results of fluorescent microsphere in our 4× and 1× zoom imaging modes with different sampling ratios of 0.2%, 0.1%, 0.05%, and 0.03%. The orange curves in the inset are the intensity profile along the corresponding orange line trace. The corresponding scale bar is 20 μm. **b** The curves of peak signal-to-noise ratio (PSNR) and structural similarity index measure (SSIM) with different sampling ratios. PAM programmable array microscope, DCCM deep compressive confocal microscope, GT ground truth, WF wide field.

We employ fluorescent microspheres^44^ of size 5 μm to validate the high-speed zoom imaging mode. Specifically, we merge the pixels of the original adaptively learned 128 × 128 pattern using a 4 × 4 sized window for SPI sampling, followed by the neighborhood unmerging patterns for jointly achieving fast neighborhood unmerging and reconstruction. Figure 3a presents the results of our zoom imaging experiments. Our method realized confocal imaging of fluorescent microspheres in 4× zoom imaging mode, with a minimum sampling ratio of only 0.03%, corresponding to the number of measurements *m* = 5. The sampling ratio required is almost three orders of magnitude lower than the conventional compressive sensing reconstruction^42^. According to the DMD refresh rate $R=15{kHz}$, the imaging frame rate $F=\frac{R}{2m}=1500$ fps in the +1/−1 sampling mode^28^, thereby providing an opportunity to observe transient life activities. In addition, the curves of PSNR and SSIM in Fig. 3b show that the 4× zoom imaging quality of the fluorescent microspheres is superior to that of the 1× zoom experiment at an arbitrary sampling ratio in [0, 0.03]. In Fig. 3a, the PNSR and SSIM scores of the fluorescent spheres reconstruction at a sampling ratio of 0.03% in 4× imaging mode are 34.74 and 0.9283, respectively. Note that the 1× zoom is the common imaging mode, which employs the 128 × 128 pattern without pixel merging.

The zoom imaging mode has been demonstrated to effectively reduce the sampling ratio compared to traditional compressive sensing imaging modes. Moreover, it can achieve image reconstruction with a minimum sampling ratio of 0.03% in our test. Meanwhile, the minimum sampling ratio required for DCCM reconstruction may be affected by different scenario sparsity and sample types. We list the reconstruction results of fluorescent microspheres for different scene sparsity in Supplementary Fig. S6. The minimum sampling ratio required for DCCM reconstruction increases with decreasing sparsity of the image. We also performed experimental analysis on different fluorescent samples in the next section.

**Fig. 4:**
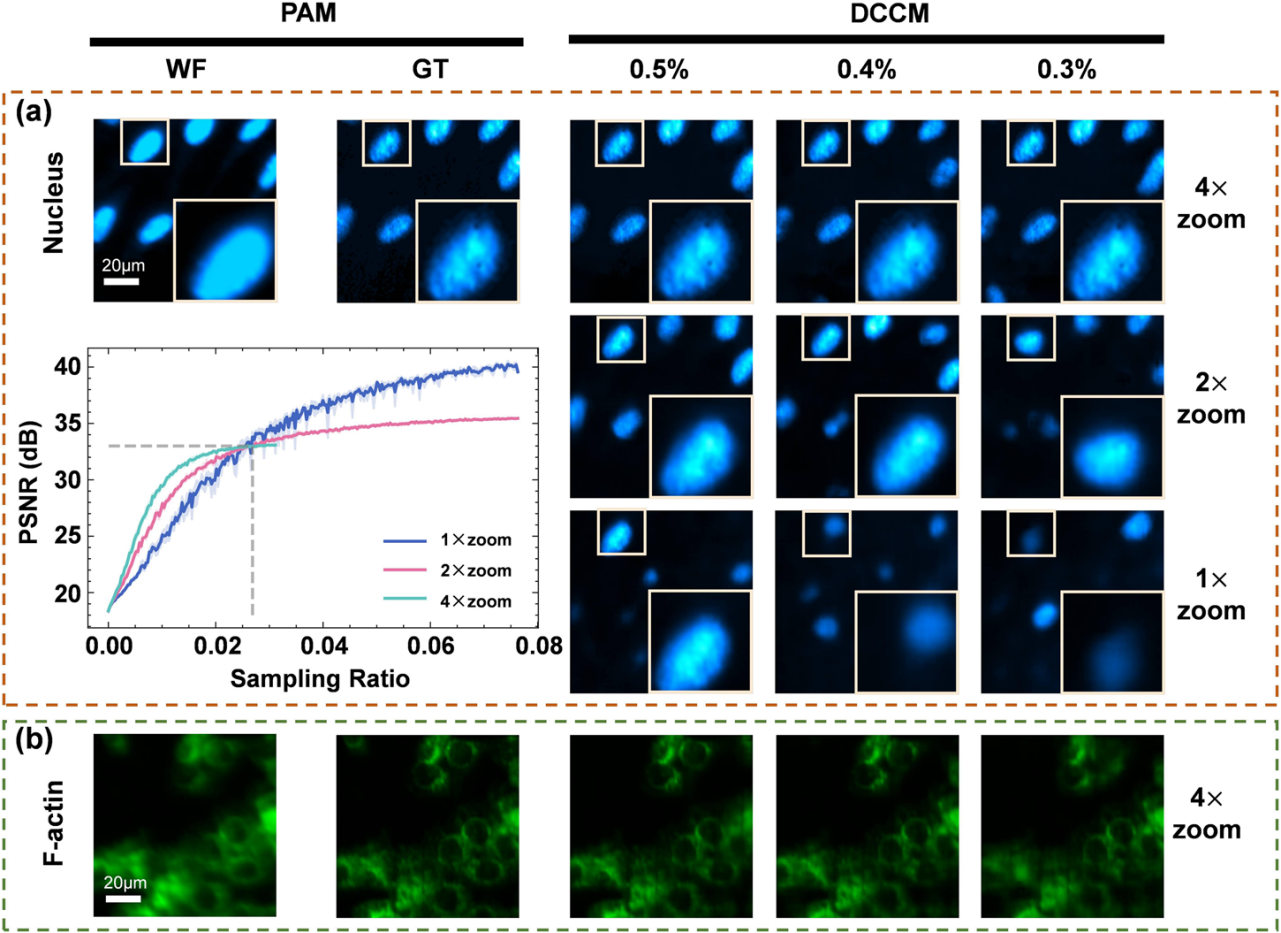
The high-speed zoom imaging for Nucleus and F-actin samples. **a** Experimental results for Nucleus in 4×, 2×, and 1× zoom imaging modes with different sampling ratios of 0.5%, 0.4%, and 0.3%. The bottom left corner shows the curves of peak signal-to-noise ratio (PSNR) scores with different sampling ratios in the inset. **b** Experimental results for F-actin at the 4× zoom imaging mode with the same sampling ratios as in (**a**). The corresponding scale bars in (**a**) and (**b**) are both 20 μm. PAM programmable array microscope, DCCM deep compressive confocal microscope, WF wide field, GT ground truth.

Furthermore, we validated and analyzed the zoom imaging mode on cell samples. We conduct experiments on Nucleus using 4× zoom, 2× zoom, and 1× zoom. The 2× zoom mode utilizes a 2 × 2 sized window to merge the original adaptively learned 128 × 128 pattern. The experimental results are shown in Fig. 4a. It can be clearly seen that the 4× zoom imaging realizes the reconstruction of confocal images at a minimum sampling ratio of 0.4% (the number of measurements *m* = 60). According to the curve of PSNR with different sampling ratios, the 4× zoom imaging mode achieves the highest PSNR scores than the 1× zoom imaging mode and the 2× zoom imaging mode when sampling ratios are below approximately 2.7%. The 4× zoom enables image reconstruction with the least amounts of measurements, while the reduced resolution of the reconstructed image is compensated by the network. Additionally, we can realize the confocal imaging (Fig. 4b) and 3D imaging (Supplementary Fig. S7) of F-actin using 4× zoom mode at a sampling rate of 0.4% (the number of measurements *m* = 60).

In addition, to validate the generalization ability of DCCM-Net for different scenarios, we also performed compressive confocal reconstruction of the potato tubers autofluorescence at ultra-low sampling ratios. The result please see Supplementary Fig. S8. As shown in the above results, the zoom imaging mode has the best performance on the fluorescent microsphere compared to the cell samples and potato tubers autofluorescence, which may be attributed to the properties of fluorescent microsphere samples, including the sparsity, high Signal-to-noise ratio (SNR), and morphological simplicity. This indicates the performance of our method in cell imaging may be further enhanced by utilizing samples with more sparse and simple structures or the markers with higher fluorescence quantum efficiency.

## Discussion

In this work, our proposed DCCM and DCCM-Net enable high-speed confocal imaging at ultra-low sampling ratios for deeply compressed image reconstruction. Experimental results demonstrate that DCCM-Net can achieve image reconstruction within 50 ms, exceeding the speeds of traditional reconstruction algorithms based on iterative optimization, which facilitates real-time image processing and analysis.

The innovative high-speed zoom imaging mode greatly reduces the sampling ratio compared to the traditional single-pixel imaging method. However, the minimum sampling ratio required for imaging is affected by the type of sample. In this paper, we performed experiments on four types of samples which are fluorescent spheres, Nucleus, F-actin, and potato tuber autofluorescence. The minimum sampling ratio for Nucleus, F-actin, and potato tuber image reconstruction is 0.4%. Moreover, we can achieve fluorescent sphere image reconstruction with a minimum sampling ratio of 0.03%. Based on the above results, we found that compared to fluorescent spheres, the other three fluorescent samples have more complex structural features and lower image sparsity, which can represent a wider range of fluorescent samples. Therefore, we believe that DCCM can perform image reconstruction at a sampling ratio of about 0.4% for conventional fluorescent samples. For samples with high sparsity and single shape, DCCM can realize a lower sampling ratio to achieve the reconstruction.

**Fig. 5:**
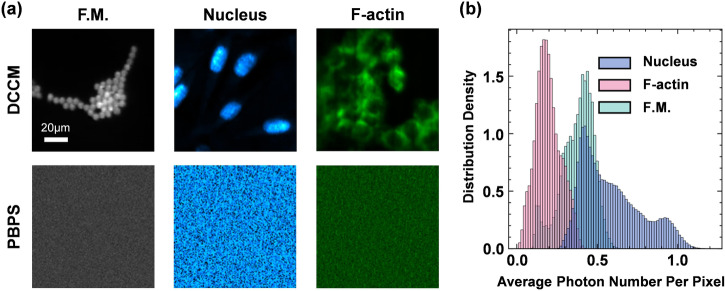
The analysis of imaging at the single-photon level. **a** The imaging results of DCCM and PBPS under the same light intensity and the same refresh rate of DMD. The corresponding scale bar is 20 μm. F.M. means Fluorescent Microspheres. **b** The probability distribution of the average number of photons per pixel detected in a single-pattern measurement. DCCM deep compressive confocal microscope, PBPS point-by-point scanning.

The DCCM has the ability to detect extremely weak signals by means of high-throughput aggregation collection via a single-photon detector PMT. Figure 5a shows that our DCCM equipped with DCCM-Net achieves high-quality confocal imaging even in extremely weak signal conditions, but the traditional point-by-point scanning (PBPS) detection method fails. In Fig. 5a, the laser power densities are only 0.09${{W\; cm}}^{-2}$, 0.4${{W\; cm}}^{-2}$, and 4${{W\; cm}}^{-2}$ for experiments on fluorescent microspheres, Nucleus, and F-actin, respectively, thus enabling low-phototoxicity observation. As depicted in Fig. 5b, we provide the probability distribution of the average number of photons per pixel detected in a single-pattern measurement for different samples. Evidently, the result in fluorescent microsphere imaging is concentrated around 0.4 photons per pixel. It is calculated by dividing the average number of photons detected in a single-pattern measurement (around 400 photons) by the number of valid pixels in the image (nearly 1000 pixels). The number of valid pixels refers to the area occupied by the sample in the image. The average photons per pixel of Nucleus and F-actin are 0.4 and 0.2, respectively. Such weak signal conditions are challenging for conventional imaging techniques, which further reveals the superiority of our DCCM.

## Conclusion

In conclusion, we develop a DCCM system along with a DCCM-Net reconstruction algorithm, which enables high-speed, low-phototoxicity confocal microscopy imaging. Based on the symmetric reflection properties of DMD, we can acquire the paired and registered datasets using our DCCM system. These datasets are used as training sets for the DCCM-Net to improve its robustness to realistic noise. Moreover, the DCCM benefits from the optimal data-driven learned modulation patterns to improve its sensing ability for SPI. The performance of such a positive feedback iterative system may be continually enhanced as the dataset increases. Especially, we innovatively proposed a high-speed zoom imaging mode based on the characteristics of our system. As a result, the DCCM achieves single-pixel confocal imaging with a sampling ratio of about 0.03%, which is almost three orders of magnitude lower than the sampling ratio required for the conventional CS method. Furthermore, it realizes single-photon level confocal imaging with no more than 0.4 photons per pixel on average in the single-pattern measurement. This reduces the excitation light power requirement, leading to low phototoxicity. We believe that DCCM would pave the way for high-speed long-duration 3D confocal imaging of living biological samples in the future. Meanwhile, by incorporating Time-Correlated Single Photon Counting and a spectroscopic prism, the DCCM can be extended to achieve fluorescence lifetime and spectral imaging, thereby enabling a wider range of multimodal biomicroscopic imaging studies.

## Methods

### System calibration

In the DCCM system, the size and resolution of the reconstructed image are determined by the pattern on the DMD since the principle of single-pixel imaging is to reconstruct the image on DMD mirrors. Moreover, the symmetrical optical paths of SPI and PAM along the DMD normal axis enable the DCCM to pair the single-pixel acquisition data with confocal images, thus it is possible to perform the pixel-by-pixel alignment between the sCMOS and DMD. To implement this, we first load the stripe pattern onto the DMD, followed by irradiating the DMD vertically with a halogen light source. Consequently, the sCMOS receives the reflective stripe pattern from the DMD. We then adjust the three-axis rotation stage of the sCMOS base to align the bright stripes parallel to the camera element columns or rows. Finally, we resize the imaging by adjusting the zoom lens focal length to ensure that the spacing and length of bright stripes captured by the sCMOS are basically consistent with the stripe pattern. However, the reflection property of the DMD micromirrors along the normal ±12° direction causes a 24° angle between the DMD micromirror array and the sCMOS detector array, leading to image distortion which is equivalent to a perspective transformation of the sCMOS array plane with respect to the DMD array plane. To address this, we use a telecentric lens (Computar TEC55 – 55 mm Telecentric Lens) to increase the depth of field and apply an affine transformation to correct the image distortion, thus ensuring pixel-by-pixel matching between the DMD and sCMOS (see Supplementary Fig. S9 for the details).

### Acquisition of ground truth for network training

To ensure the reconstruction with high quality, the confocal images captured by our DCCM are utilized as the ground truth for the training of DCCM-Net. Due to the programmable properties of the DMD, the DCCM can offer two confocal imaging schemes for samples with different fluorescence intensities. The first is a single-exposure confocal imaging scheme, which is suitable for samples with high fluorescence quantum yield, such as fluorescent spheres. In this scheme, the exposure time of sCMOS is the accumulation of different multi-point scanning patterns of DMD. The second is a multi-exposure stack scanning scheme for samples with weak fluorescence. This scheme requires simultaneous triggering of the camera exposure and DMD. The final confocal image is the stack of images with different multi-line patterns. In addition, the pixel alignment between sCMOS and DMD ensures that the confocal image and the single-pixel compressed data are paired and registered. Notably, the DCCM can switch freely between wide-field, SPI, and confocal imaging as required. More information on the confocal image acquired by PAM can be found in Supplementary Fig. S10.

### The deep SPI reconstruction algorithm

Mathematically, the imaging task of DCCM is to infer the object intensity $x\in {{\mathbb{R}}}^{N}$ from its low-dimensional single-pixel measurement $y\in {{\mathbb{R}}}^{M}$ under noisy acquisition $y={Ax}+n$, where $N=H\times W$ and *M* are the sizes of the target image and observed measurement, the linear projection is achieved by sampling patterns $A\in {{\mathbb{R}}}^{M\times N}$, $n\in {{\mathbb{R}}}^{M}$ is the observation noise, and the sampling ratio is defined as $r=\frac{M}{N}$. In DCCM, the extremely small value $r$ with $M\ll N$ brings not only the benefits of sampling cost reduction (e.g. sampling acceleration and energy saving), but also the difficulty of predicting $x$ from only $y$ and $A$, since it becomes a seriously ill-posed problem without other information. In practice, two fundamental issues of CS imaging are the design of sampling patterns and the development of reconstruction algorithms^45^, which are resolved by our proposed learnable bipolar patterns and deep unrolled DCCM-Net.

To enhance the sensing ability and facilitate our optical implementation, we propose to learn bipolar sampling pattern $A$ from some pre-collected ground truths of microscopic intensity. Specifically, we obtain 1000 images of a different Nucleus sample from our confocal imaging branch, introduce a learnable auxiliary variable $\varPhi \in {{\mathbb{R}}}^{M\times N}$ and utilize the following ${{\ell}}_{2}$ loss function to optimize $\varPhi$ with given ground truth $x$:

1 $${{{{{{\mathscr{L}}}}}}}_{{{{{{\rm{matrix}}}}}}}\left(\varPhi \right)=\frac{1}{N}{{{{{{\rm{||}}}}}}{A}^{{{{{{\rm{T}}}}}}}{Ax}-x{{{{{\rm{||}}}}}}}_{2}^{2},\,A=\frac{1}{\sqrt{N}}{{{{{\rm{Sign}}}}}}(\varPhi ).$$

Here, the mean squared error between $x$ and the initial estimation ${A}^{T}y$ of its simulated CS measurement ${Ax}$ is employed as a metric of the information-preserving ability of $A$ for microscopic image domains. The bipolar function ${{{{{\rm{Sign}}}}}}\left(\cdot \right)$ maps all the non-negative pattern elements to +1 and the others to −1. Its derivative is set to 1 for ensuring the differentiability in back-propagation. After our training by ${{{{{{\mathscr{L}}}}}}}_{{{{{{\rm{matrix}}}}}}}$ in Eq. (1), the adaptively learned sampling patterns are generated from $\varPhi$ and then adopted in the single-pixel imaging branch of DCCM. And one can randomly select $m$ rows ($m\le M$) from the learnable $A$ to obtain new sampling patterns with ratio $\frac{m}{N}$.

The CS imaging task in DCCM can be formulated as the following regularized optimization problem:

2 $${\min }_{x}\frac{1}{2}{{{{{{\rm{||}}}}}}{Ax}-y{{{{{\rm{||}}}}}}}_{2}^{2}+\lambda {{{{{\rm{R}}}}}}(x),$$

where ${{{{{\rm{R}}}}}}:\,{{\mathbb{R}}}^{N}{\mathbb{\to }}{\mathbb{R}}$ is a regularization function assumed to be convex with the weighting factor $\lambda \in {{\mathbb{R}}}^{+}$. To exploit both the merits of traditional optimization frameworks and neural networks, we adopt the proximal gradient descent (PGD) algorithm^39^ as the basic framework to guide our architecture design of DCCM-Net. Specifically, given an initialization ${\hat{x}}^{(0)}$, PGD solves the CS problem in Eq. (2) by iterating between the following two update steps:

3 $${z}^{\left(k\right)}={\hat{x}}^{\left(k-1\right)}-\rho {A}^{{{{{{\rm{T}}}}}}}\left(A{\hat{x}}^{\left(k-1\right)}-y\right)\,\in {{\mathbb{R}}}^{N},$$

4 $${\hat{x}}^{\left(k\right)}={{{{{{\rm{prox}}}}}}}_{\lambda {{{{{\rm{R}}}}}}}\left({z}^{\left(k\right)}\right)={{{{{{\rm{argmin}}}}}}}_{x}\frac{1}{2}{{{{{{\rm{||}}}}}}x-{z}^{\left(k\right)}{{{{{\rm{||}}}}}}}_{2}^{2}+\lambda {{{{{\rm{R}}}}}}(x)\,\in {{\mathbb{R}}}^{N},$$

where $k$ and $\rho$ denote the iteration index and step size, respectively. Equation (3) is the gradient descent step, while Eq. (4) is the proximal mapping step that is critical to solving Eq. (2) with prior knowledge of microscopic images^46^.

As Fig. 1b illustrates, our DCCM-Net $F$ is composed of two cascaded sub-networks: an initialization subnet (IS) ${F}_{{{{{{\rm{IS}}}}}}}$ and a deep PGD-unrolling RS ${F}_{{{{{{\rm{RS}}}}}}}$. The reconstruction is achieved by $\hat{x}=F(y,A,r)={F}_{{{{{{\rm{RS}}}}}}}({F}_{{{{{{\rm{IS}}}}}}}(y,A),y,A,r)$. Given a single-pixel measurement $y$, in our IS, the initial intensity estimation is obtained by ${\hat{x}}_{{{{{{\rm{init}}}}}}}={F}_{{{{{{\rm{IS}}}}}}}\left(y,A\right)={A}^{{{{{{\rm{T}}}}}}}y$, which efficiently addresses the dimension mismatch between the measurement domain (${{\mathbb{R}}}^{M}$) and image domain (${{\mathbb{R}}}^{N}\,$ or ${{\mathbb{R}}}^{H\times W}$) without introducing extra parameters. In RS, to make physics information of $y$, $A$, and $r$ available to be sufficiently utilized and adaptively fused in harmony, we propose to obtain the shallow feature of our initialization by an extraction module ${F}_{{{{{{\rm{ext}}}}}}}$, dynamically conditioned by the sampling ratio, then refine the feature through $K$ PGD stage modules ${F}_{{{{{{\rm{stage}}}}}}}^{(k)}\,(k={{{{\mathrm{1,2}}}}},\cdots ,K)$ by mapping Eqs. (3) and (4) to deep network components, and finally transform the enhanced feature to image domain by a recovery module ${F}_{{{{{{\rm{rec}}}}}}}$. Formally, our RS can be expressed as $\hat{x}={F}_{{{{{{\rm{RS}}}}}}}({\hat{x}}_{{{{{{\rm{init}}}}}}},y,A,r)={F}_{{{{{{\rm{rec}}}}}}}(\cdots {F}_{{{{{{\rm{stage}}}}}}}^{\left(k\right)}(\cdots {F}_{{{{{{\rm{ext}}}}}}}({\hat{x}}_{{{{{{\rm{init}}}}}}},r)\cdots ,y,A)\cdots )$. The concrete structure design of our RS and more details about our DCCM-Net are given in Supplementary Fig. S2.

As described above, our adaptive bipolar sampling patterns and DCCM-Net can be implemented and learned accordingly. Concretely, the trainable parameter sets for $A$ and DCCM-Net are expressed as ${\varTheta }_{{{{{{\rm{matrix}}}}}}}=\{\varPhi \}$ and ${\varTheta }_{{{{{{\rm{DCCM}}}}}}-{{{{{\rm{Net}}}}}}}=\{{F}_{{{{{{\rm{ext}}}}}}},{F}_{{{{{{\rm{rec}}}}}}}\}\cup {\{{F}_{{{{{{\rm{stage}}}}}}}^{\left(k\right)}\}}_{k=1}^{K}$, respectively. We first train ${\varTheta }_{{{{{{\rm{matrix}}}}}}}$ by ${{{{{{\mathscr{L}}}}}}}_{{{{{{\rm{matrix}}}}}}}$ in Eq. (1) and then employ the learned $A$ to capture the registered data pairs by our two optical acquisition branches to form a dataset ${\{({y}_{i},{x}_{i})\}}_{i=1}^{l}$. For a given data pair $(y,x)$, we use the following ${\ell}_{1}$ loss function to optimize all the parameters in ${\varTheta }_{{{{{{\rm{DCCM}}}}}}-{{{{{\rm{Net}}}}}}}$ indiscriminately:

5 $${{{{{{\mathscr{L}}}}}}}_{{{{{{\rm{DCCM}}}}}}-{{{{{\rm{Net}}}}}}}\left({\varTheta }_{{{{{{\rm{DCCM}}}}}}-{{{{{\rm{Net}}}}}}}\right)=\frac{1}{N}{{{{{{\rm{||}}}}}}F(y,A,r)-x{{{{{\rm{||}}}}}}}_{1}.$$

To improve the data diversity and make our DCCM-Net scalable to arbitrary sampling ratios in ${\{\frac{m}{N}\}}_{m=1}^{M}$, we develop a random data augmentation mechanism for training enhancement. Specifically, given a training data pair $\left(y,x\right)$, we randomly select a measurement number $m$ from $\{1,\cdots ,M\}$ and then randomly select $m$ elements from $y$ and the corresponding $m$ rows from $A$ to form a new pair $\left({y}^{{\prime} },x\right)$ with patterns ${A}^{{\prime} }$ and sampling ratio ${r}^{{\prime} }=\frac{m}{N}\le r$. It is verified to be effective by our experiments with the theoretical potential of generating $\left({2}^{M}-1\right)$ data pairs with various levels of reconstruction difficulties from an original single pair $\left(y,x\right)$ of ratio $r=\frac{M}{N}$.

In the high-speed zoom imaging mode, the adaptively learned sampling patterns are downscaled by average pooling and then bipolarized using ${{{{{\rm{Sign}}}}}}\left(\cdot \right)$ for our neighborhood merging sampling. For the corresponding deep neighborhood unmerging reconstruction, we replace the original sampling patterns for DCCM-Net with the nearest upscaled new ones to obtain the ultra-ratio-enhanced network without introducing structural modifications.

In our implementation, we set image size $N=H\times W=16384$ with $H=W=128$ and maximal measurement size $M=5000$. For DCCM-Net, we set the stage number $K=9$ and feature channel number $C=128$ by default. For each sample, we collect $l=1200$ data pairs and randomly select 50 pairs to construct the test set, and collect the other 1150 pairs to form the training set. The batch size is set to $B=16$. Our learnable bipolar sampling patterns $A$ and DCCM-Net are both implemented in PyTorch^47^ and separately trained as above described by the Adam^48^ optimizer with a momentum of 0.9 and a weight decay of 0.999. It takes about a week in total to learn sampling patterns and a DCCM-Net on an NVIDIA RTX 4090 GPU with $1.5\times {10}^{6}$ iterations. The learning rate is initialized to $2\times {10}^{-4}$ and finally decayed to $2\times {10}^{-6}$.

Peak signal-to-noise ratio (PSNR) and structural similarity index measure (SSIM)^49^ are employed as the metrics for all the quantitative evaluations. We plot the variation of PSNR and SSIM values with sampling ratio, respectively. To assess the performance at a specific sampling ratio $M/N$ ($M={{{{\mathrm{1,2}}}}},...,5000$), we adopt a randomized selection strategy. In our study, for each test image of size 128$\times$128, we initially collected 5000 measurements using our learned sampling patterns $A$. From the 5000 measurements, we randomly select $M$ observation elements to form a new observation vector ${y}^{\prime} .$ Simultaneously, we select corresponding rows from the original sampling matrix to create a new sampling pattern ${A}^{\prime}$, aligning with the selected $M$ measurement elements. This process ensures that ${y}^{\prime} \,={A}^{\prime} x$ holds under ideal conditions. For reconstruction at the given sampling ratio $M/N$, we employ ${A}^{\prime}$ to reconstruct an estimate of $x$ from ${y}^{\prime}$, applying this method uniformly across all images in our test set. The PSNR and SSIM for each image are calculated by comparing the reconstructed image, obtained from ${y}^{\prime}$ using the new sampling pattern ${A}^{\prime}$ and our DCCM-Net, against the original image $x.$ This approach allows us to compute the PSNR and SSIM for each image under the specified sampling conditions. The average PSNR and SSIM values across the test set provide a comprehensive evaluation of our reconstruction quality at the given sampling ratio.

### Cell culture and staining

The NIH3T3 cells and MC38 cells are maintained in RPMI1640 medium supplemented with 10% FBS, and 1% pen/strep antibiotics (all from GIBCO). Prior to staining, a total of 10,000 cells are seeded on 18 mm diameter round coverslips and returned to the CO_2_ incubator for 24 h. Following this, the cells are fixed with 4% paraformaldehyde in 1× PBS for 15 min at room temperature and washed twice with 1× PBS. After that, the cells are permeated with 0.2% TritonX-100 supplemented with 3% BSA in 1× PBS for 30 min at room temperature. The Tom20 antibody (rabbit-anti-mouse, from Santa Cruz) is 500× diluted into 3% BSA and applied to the permeated cell at 4 °C overnight for mitochondria labeling. On the following day, the cells are washed 5 times with 3% BSA and incubated for 2 h at room temperature with 200× diluted Alexa flour-568 labeled goat-anti-rabbit antibody for mitochondrial fluorescent staining. After that, the cells are washed 5 times with 3% BSA, and the cells are incubated for 30 min at room temperature with 100× diluted AlexaFluor-488 phalloidin for F-actin fluorescent staining. Following this, the cells are washed 5 times with 1× PBS, counterstained with 2 μg mL^−^^1^ DAPI, 1× PBS washed, and mounted on glass slides with AntiFade mounting medium (from Thermo Fisher). The prepared samples are then ready for imaging.

### Reporting summary

Further information on research design is available in the Nature Portfolio Reporting Summary linked to this article.

### Supplementary information

Supplementary Information

Reporting Summary

## Data Availability

Data underlying the results presented in this paper are not publicly available but may be obtained from the authors upon reasonable request.

## Code availability

For reproducible research, the complete source code of DCCM-Net can be obtained from the following publicly accessible resource^50^.
