# Supplementary material for: Compressive confocal microscopy imaging at the single-photon level with ultra-low sampling ratios
Source: Commun Eng. 2024 Jun 25;3:88. doi: 10.1038/s44172-024-00236-x (PMC11199654; doi:10.1038/s44172-024-00236-x)
Supplement: Supplementary file 1 — Supplementary Information [file 44172_2024_236_MOESM1_ESM.pdf]

# Supplementary Information

# Compressive confocal microscopy imaging at the single-photon level with ultra-low sampling ratios

Shuai Liu[1,*], Bin Chen[2,*], Wenzhen Zou[3], Hao Sha[3], Xiaochen Feng[3], Sanyang Han[1], Xiu Li[1], Xuri Yao[4], Jian Zhang[2,†], Yongbing Zhang[3,†]

[1]Tsinghua Shenzhen International Graduate School, Tsinghua University, Shenzhen 518055, China

[2]School of Electronic and Computer Engineering, Peking University Shenzhen Graduate School, Shenzhen, Guangdong 518055, China

[3]School of Computer Science and Technology, Harbin Institute of Technology (Shenzhen), Shenzhen, Guangdong 518055, China

[4]Center for Quantum Technology Research, School of Physics, Beijing Institute of Technology, Beijing 100081, China

[*]These authors contributed equally

[†]J.Z.(zhangjian.sz@pku.edu.cn), Y.Z.(ybzhang08@hit.edu.cn)

**Supplementary Note 1.**

**The reconstruction comparisons of DCCM-Net trained with realistic datasets and simulated datasets**

One of the biggest advantages of DCCM is the ability to obtain paired training datasets. This allows the network to be robust against noise in real scenes by using real datasets to train the network. We used realistic datasets and simulated datasets to train the network respectively to compare the reconstruction performance. The simulated compressed data $y$ is obtained by multiplying the ground truth $x$ with the sampling matrix $A$. Fig. S1 shows the test results of the DCCM-Net on real compressed data obtained by DCCM after training with realistic and simulated datasets, respectively. It can be clearly seen that the network trained with real data has better reconstruction performance.

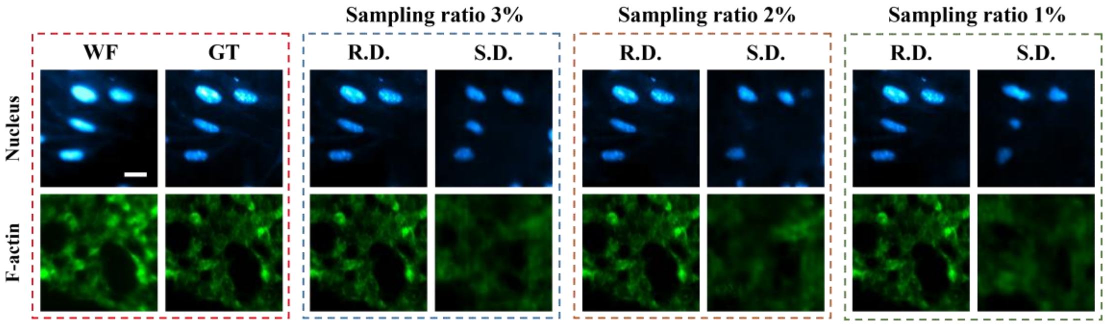


**Figure S1**. **The reconstruction of DCCM-Net trained with realistic datasets and simulated datasets.** R.D.: realistic datasets, S.D.: simulated datasets. The corresponding scale bar is 20 $\mu m$. WF: wide field; GT: ground truth.

**Supplementary Note 2.**

**Structural details of the recovery subnet (RS) of our proposed DCCM-Net**

As illustrated in Fig. S2, given the initial estimation $\hat{x}_{\text{init}} = F_{\text{IS}}(y, A) = A^T y \in \mathbb{R}^N$ from initialization subnet (IS) $F_{\text{IS}}$, the sampling ratio $r \in [0,1]$, and the sampling patterns $A \in \mathbb{R}^{M \times N}$, our RS ($F_{\text{RS}}$) generates a channel-wise concatenation of $\hat{x}_{\text{init}}$ and sampling ratio map $M \in \mathbb{R}^{H \times W}$ with all elements being $r$. Subsequently, a $3 \times 3$ convolution layer and a PixelUnshuffle [1] of scaling factor $s$ are applied, followed by $K$ PGD [2] -unrolled stages to extract the shallow image feature of $C$ channels, refining it in a step-by-step manner. The final recovered result $\hat{x}$ is obtained by a $3 \times 3$ convolution layer and a PixelShuffle of scaling factor $s$ from the refined feature. The structural details of our RS design are given as follows.

Considering the trade-off among the capacity, receptive field, and computation cost of the network, our $F_{\text{ext}}$ employs a convolution, followed by a PixelUnshuffle $(\cdot)\downarrow_s$ with factor $s$, to fuse the reshaped $\hat{x}_{\text{init}}$ of size $1 \times H \times W$ and a uniform sampling ratio map $M = [r]_{1 \times H \times W}$ to generate a downscaled feature as:

$$\hat{X}^{(0)} = F_{\text{ext}}(\hat{x}_{\text{init}}, r) = \left(Conv_{\text{ext}}([\hat{x}_{\text{init}}, M])\right)\downarrow_s \in \mathbb{R}^{C \times \frac{H}{s} \times \frac{W}{s}}, \quad (1)$$

where $[\hat{x}_{\text{init}}, M] \in \mathbb{R}^{2 \times H \times W}$ is the channel-wise concatenation of $\hat{x}_{\text{init}}$ and $M$.

Each unrolled PGD stage includes a gradient descent module and a proximal mapping module in series. The former performs the analytic gradient descent process on the first $s^2$ feature channels while keeping the other $(C - s^2)$ channels unchanged to hold a high-throughput transmission [3] without information loss. The latter then employs two cascaded nonlinear activation-free blocks (NAFBs) to carry out a denoising process. Specifically, in the $k$-th unrolled stage module $F_{\text{stage}}^{(k)}$, we map Eqs. (3) and (4) in the Methods to two cascaded refinement steps. To maintain a maximized information flow and enhance the network's flexibility, we perform the gradient descent in Eq. (3) in the Methods on the front feature part $\hat{X}_{:s^2}^{(k-1)}$ of shape $s^2 \times \frac{H}{s} \times \frac{W}{s}$ from $\hat{X}^{(k-1)}$ with a learnable step size $\rho^{(k)}$ and keep its latter part $\hat{X}_{s^2:}^{(k-1)}$ of $(C - s^2)$ channels unchanged. Formally, our generalized gradient descent step can be expressed as:

$$Z^{(k)} = \left[\left(\hat{x}^{(k-1)} - \rho^{(k)} A^{\mathrm{T}}\left(A\hat{x}^{(k-1)} - y\right)\right)\downarrow_s, \hat{X}_{s^2:}^{(k-1)}\right] \in \mathbb{R}^{C \times \frac{H}{s} \times \frac{W}{s}}, \hat{x}^{(k-1)} = \left(\hat{X}_{:s^2}^{(k-1)}\right)\uparrow_s \in \mathbb{R}^{1 \times H \times W}, \quad (2)$$

where $(\cdot)\uparrow_s$ is the PixelShuffle of scaling factor $s$ and $(\cdot)\downarrow_s$ is the inverse of PixelUnshuffle. The second step of $F_{\text{stage}}^{(k)}$ is a generalized proximal mapping (or a feature-level denoising step), which is achieved by our developed two cascaded basic NAFBs and can be formulated as:

$$\hat{X}^{(k)} = \text{NAFB}_2^{(k)}\left(\text{NAFB}_1^{(k)}\left(Z^{(k)}\right)\right) \in \mathbb{R}^{C \times \frac{H}{s} \times \frac{W}{s}}. \quad (3)$$

In each NAFB, the input image feature is first passed through a layer normalization (LN) [4] and transformed into a $2C$-channel feature space via an $1 \times 1$ convolution layer followed by a $3 \times 3$ depth-wise convolution layer. The feature is then evenly split into two $C$-channel parts, which are merged into a $C$-channel one by element-wise multiplication. After that, the merged feature is transformed by a $3 \times 3$ convolution layer and scaled by the attentive weights generated from another $3 \times 3$ convolution layer, obtaining the final feature residual and the output. To summarize, each NAFB contains a LayerNorm, four convolutions, and two element-wise multiplications to adaptively learn a non-linear residual for the input feature without activations. Note that a common alternative of NAFB is the classic residual block (RB), composed of a ReLU activation sandwiched by two convolutions with an identity skip connection, and we empirically find that our NAFB, improved upon the baseline transform block, is effective to bring satisfactory quality with low cost on parameters and computation, and is competent to be a better choice than RBs. By merging Eqs. (2) and (3) into one single stage module, our $F_{\text{stage}}^{(k)}$ can be expressed as:

$$\begin{aligned} \hat{X}^{(k)} &= F_{\text{stage}}^{(k)}\left(\hat{X}^{(k-1)}, y, A\right) \in \mathbb{R}^{C \times \frac{H}{s} \times \frac{W}{s}} \\ &= \text{NAFB}_2^{(k)}\left(\text{NAFB}_1^{(k)}\left(\left[\left(\left(\hat{X}_{:s^2}^{(k-1)}\right)\uparrow_s - \rho^{(k)} A^{\mathrm{T}}\left(A\left(\hat{X}_{:s^2}^{(k-1)}\right)\uparrow_s - y\right)\right)\downarrow_s, \hat{X}_{s^2:}^{(k-1)}\right]\right)\right). \end{aligned} \quad (4)$$

Finally, our recovery module $F_{\text{rec}}$ employs a convolution, followed by a PixelShuffle $(\cdot)\uparrow_s$ to reconstruct the

intensity (or image) $\hat{x}$ from the final feature $\hat{X}^{(K)}$ refined and generated by our unrolled $K$ PGD stages as:

$$\hat{x} = F_{\text{rec}}\left(\hat{X}^{(K)}\right) = \left(\text{Conv}_{\text{rec}}\left(\hat{X}^{(K)}\right)\right)\uparrow_s \in \mathbb{R}^{1\times H\times W}. \quad (5)$$

Compared to other existing deep reconstruction networks, our RS enjoys three significant advantages. First, it is optimization-inspired and has a well-defined architecture based on the traditional PGD, while maintaining a high-capacity feature-level information flow throughout the network trunk, leading to an excellent balance between its performance and interpretability. Second, it adapts to arbitrary sampling ratios learned once with our training scheme. Third, the design of NAFB, without incorporating any activation function, is proven to be more powerful and efficient than other traditional basic blocks [5], such as the classic residual block. Furthermore, it is validated to be effective and suitable for compressive confocal microscopic imaging tasks.

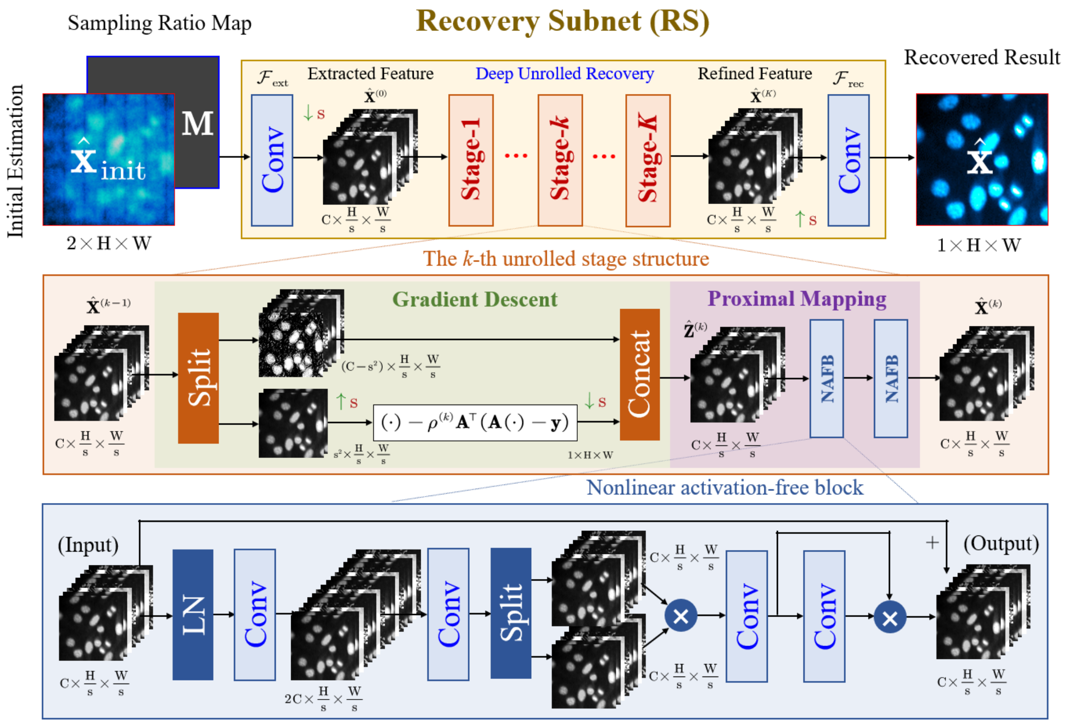


**Figure S2**. **The illustration of structural details of deep unrolled recovery subnet in our DCCM-Net.** s: scaling factor; $F_{\text{ext}}$: extraction module; $F_{\text{rec}}$: recovery module; NAFB: nonlinear activation-free block; Conv: Convolution; LN: layer normalization.

**Supplementary Note 3.**

**The experimental results of fluorescent microsphere using different sampling pattern.**

In this study, the single-pixel sampling pattern we used was generated by the network based on the features of samples, called learnable patterns. To verify the sampling performance of the learnable pattern, we provide a comparison experiment with the Hadamard pattern. Fig. S3 shows that DCCM can reconstruct the confocal image of the fluorescent microsphere even at the sampling ratio of 0.1% using the learnable pattern sampling. However, DCCM employing the Hadamard pattern is unable to reconstruct the fluorescent microsphere image below the sampling ratio of 0.3%. In addition, the imaging quality by learnable pattern is higher than that of the Hadamard pattern at any sampling ratios, as shown in the curves of PSNR and SSIM with varying sampling ratios. In particular, the PSNR and SSIM values of the learnable pattern show a faster growth trend at lower sampling ratios.

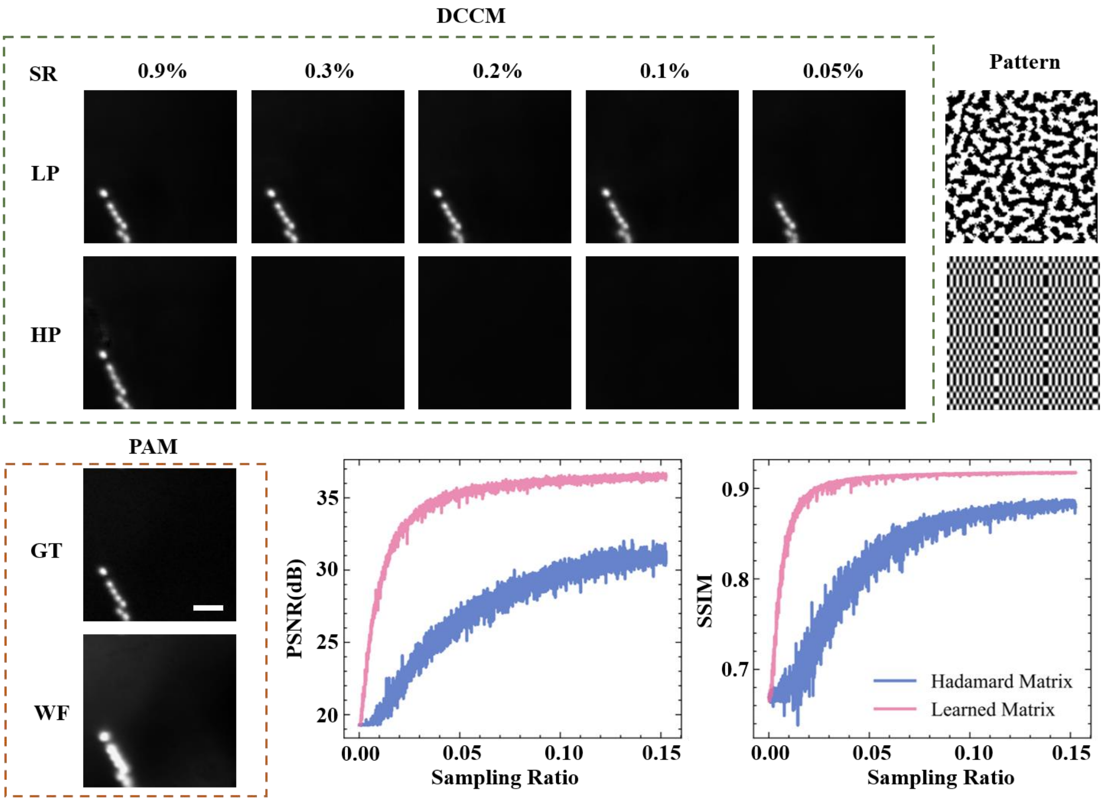


**Figure S3**. **The result comparison with different patterns over fluorescent microsphere.** The corresponding scale bar is 20 $\mu m$. DCCM: deep compressive confocal microscope; SR: sampling ratios; LP: learnable pattern; HP: Hadamard pattern; PAM: programmable array microscopy; GT: ground truth; WF: wide field; PSNR: peak signal-to-noise ratio; SSIM: structural similarity index measure.

**Supplementary Note 4.**

**The reconstruction of the Nucleus and F-actin at different sampling ratios**

Fig. S4 shows the reconstruction of the Nucleus and F-actin at more sampling ratios compared to Fig. 2.

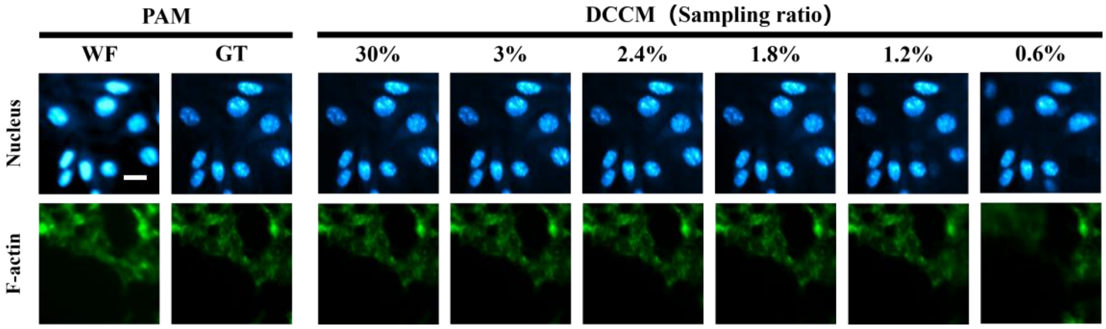


**Figure S4**. **The reconstruction of the Nucleus and F-actin at different sampling ratios.** The corresponding scale bar is 20 $\mu m$. PAM: programmable array microscopy; DCCM: deep compressive confocal microscope; WF: wide field; GT: ground truth.

**Supplementary Note 5.**

**The reconstruction of DCCM-Net and U-Net with the same amount of training datasets**

The DCCM-Net is flexible and scalable for arbitrary sampling ratios. During the DCCM training, each data pair is augmented by randomly selecting some sets of compressed measurement elements and their corresponding sampling patterns to create new data pairs with the original ground truth (GT). Hence, the DCCM can guarantee the image reconstruction quality at multiple sampling ratios after only training once. However, the traditional U-Net network is not scalable for arbitrary sampling ratios after training once. Fig. S5 shows the reconstruction results after training DCCM and U-Net respectively by using the training dataset with 2% sampling ratio. It can be clearly seen that U-Net can only reconstruct the data with a compression ratio of 2%, but not 1% and 0.5%.

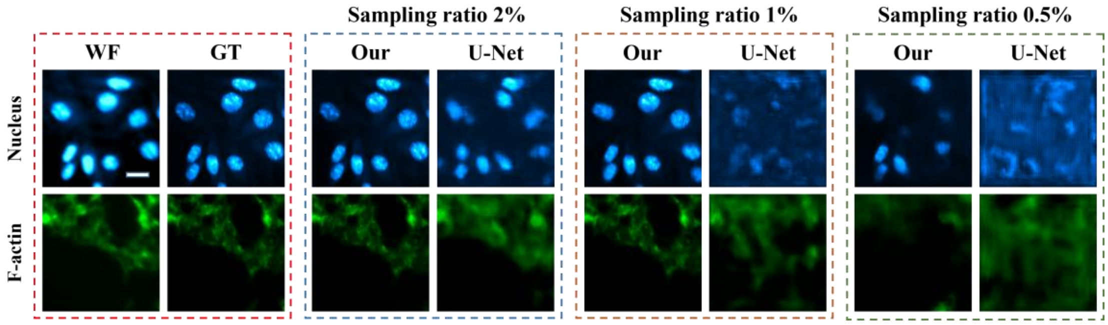


**Figure S5**. **The reconstruction of the DCCM-Net and U-Net with the same amount of training datasets.** The corresponding scale bar is 20 $\mu m$. WF: wide field; GT: ground truth.

**Supplementary Note 6.**

**Effect of image sparsity on the minimum sampling ratio required for DCCM reconstruction.**

We found that the minimum sampling ratio required for DCCM reconstruction is related to the sparsity of the image in different scenes. DCCM reconstructs four scenes of fluorescent spheres with different sparsity levels in the same measurement environment. The minimum sampling ratio required for reconstruction increases with decreasing sparsity, as shown in Fig. S6. At present, DCCM can reconstruct an image with the minimum sampling ratio of 0.03% in a specific sparse scene. Meanwhile, DCCM can reconstruct most scenes of fluorescent spheres at a sampling ratio of 0.15% in 4× zoom imaging mode. We evaluate the image quality by comparing the reconstructed image with the GT. The PSNR and SSIM values of the reconstructed images in 4× zoom imaging mode are higher than 30 and 0.9, respectively, and there are no visually obvious reconstruction errors.

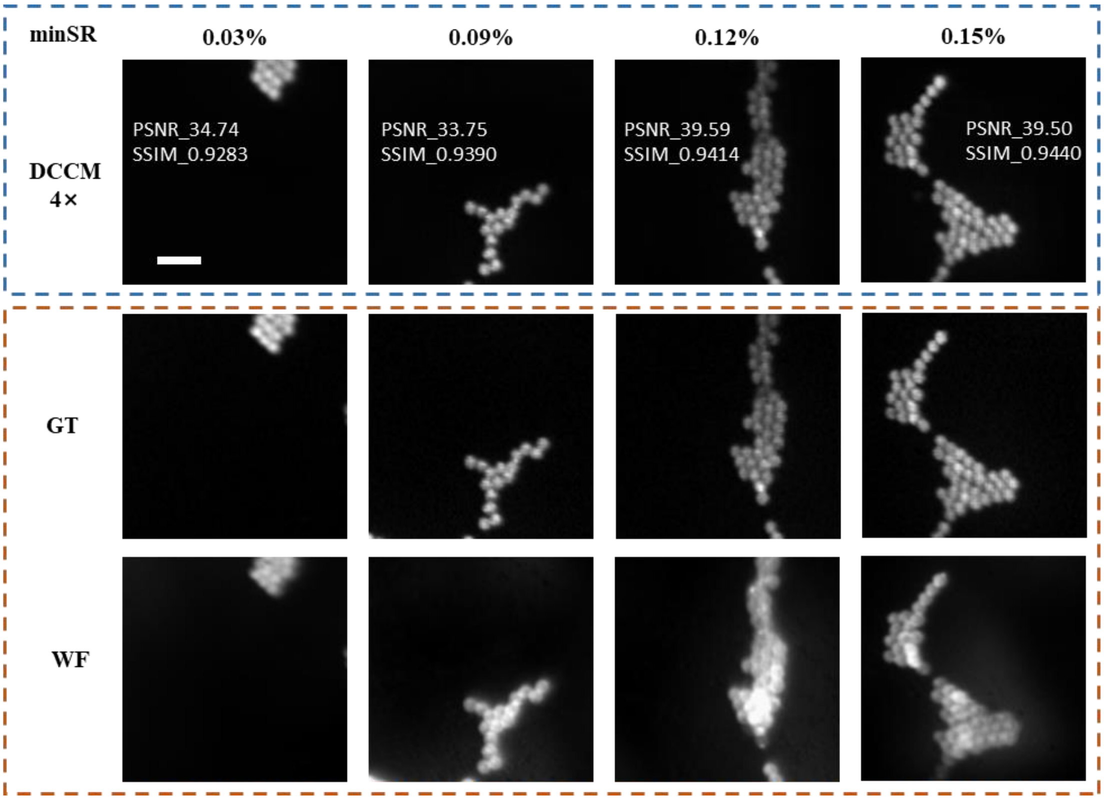


**Figure S6**. **Effect of image sparsity on the minimum sampling ratio required for deep compressive confocal microscope (DCCM) reconstruction.** The corresponding scale bar is 20 $\mu m$. minSR: minimum sampling ratios. GT: ground truth. WF: wide field; PSNR: peak signal-to-noise ratio; SSIM: structural similarity index measure.

**Supplementary Note 7.**

**The 3D imaging of F-actin samples using 4× and 1× zoom imaging mode**

To better evaluate the performance of the DCCM, we applied the zoom imaging mode to image the F-actin in 3D with 31 z slices and the adjacent optical-sections are $0.5\mu m$ away from each other in the z-direction. Fig. S7 shows the results, and it can be clearly seen that the GT has a higher resolution compared to WF, no matter in the x-y, x-z, and y-z sections. Meanwhile, the reconstruction results of DCCM are basically the same as GT at the sampling ratio of 0.4% in 4× zoom imaging mode.

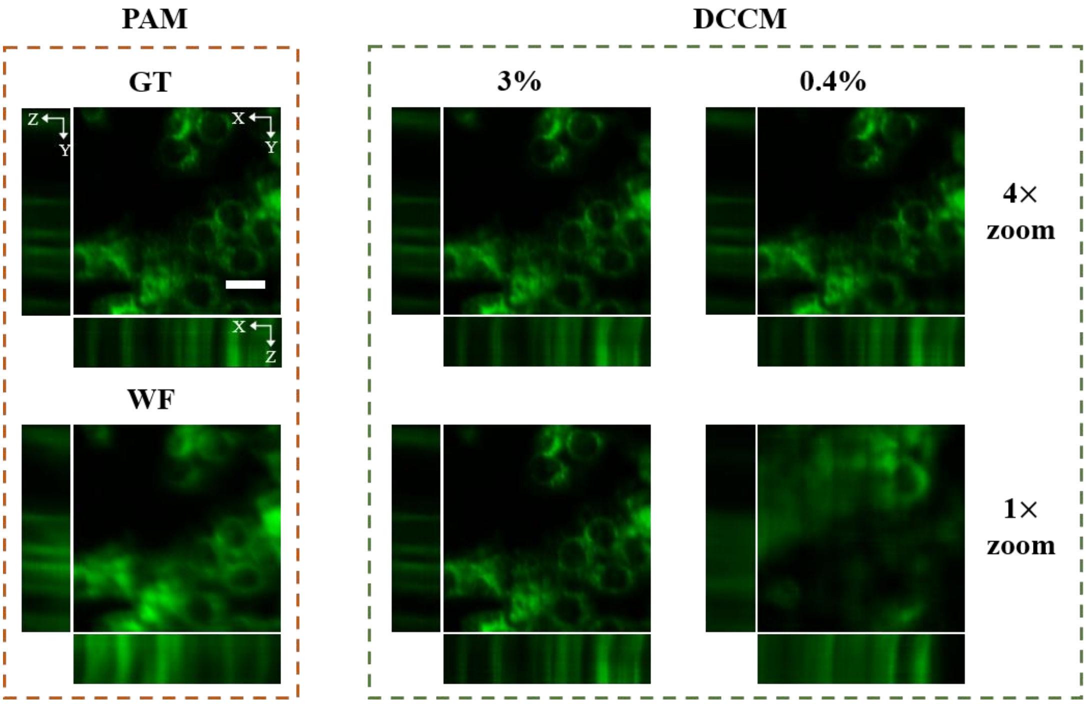


**Figure S7**. **The 3D imaging of F-actin samples using 4× and 1× zoom imaging mode.** The corresponding scale bar is 20 $\mu m$. PAM: programmable array microscopy; DCCM: deep compressive confocal microscope; GT: ground truth; WF: wide field.

**Supplementary Note 8.**

**Compressive reconstruction of potato tuber autofluorescence at ultra-low sampling ratios.**

To better evaluate the generalization ability of the DCCM-Net, we applied the zoom imaging mode to image the potato tuber autofluorescence. Fig. S8(a) shows the results, and it can be clearly seen that the GT has a higher resolution compared to WF. Meanwhile, the reconstruction results are basically the same as GT in 4× zoom imaging mode, with the sampling ratio of 0.4%, corresponding to the number of measurements $m = 60$. In addition, the curves of PSNR and SSIM in Fig. S8(b) show that the 4× zoom imaging quality of potato tuber autofluorescence is superior to that of the 1× zoom experiment at an arbitrary sampling ratio in [0, 0.03].

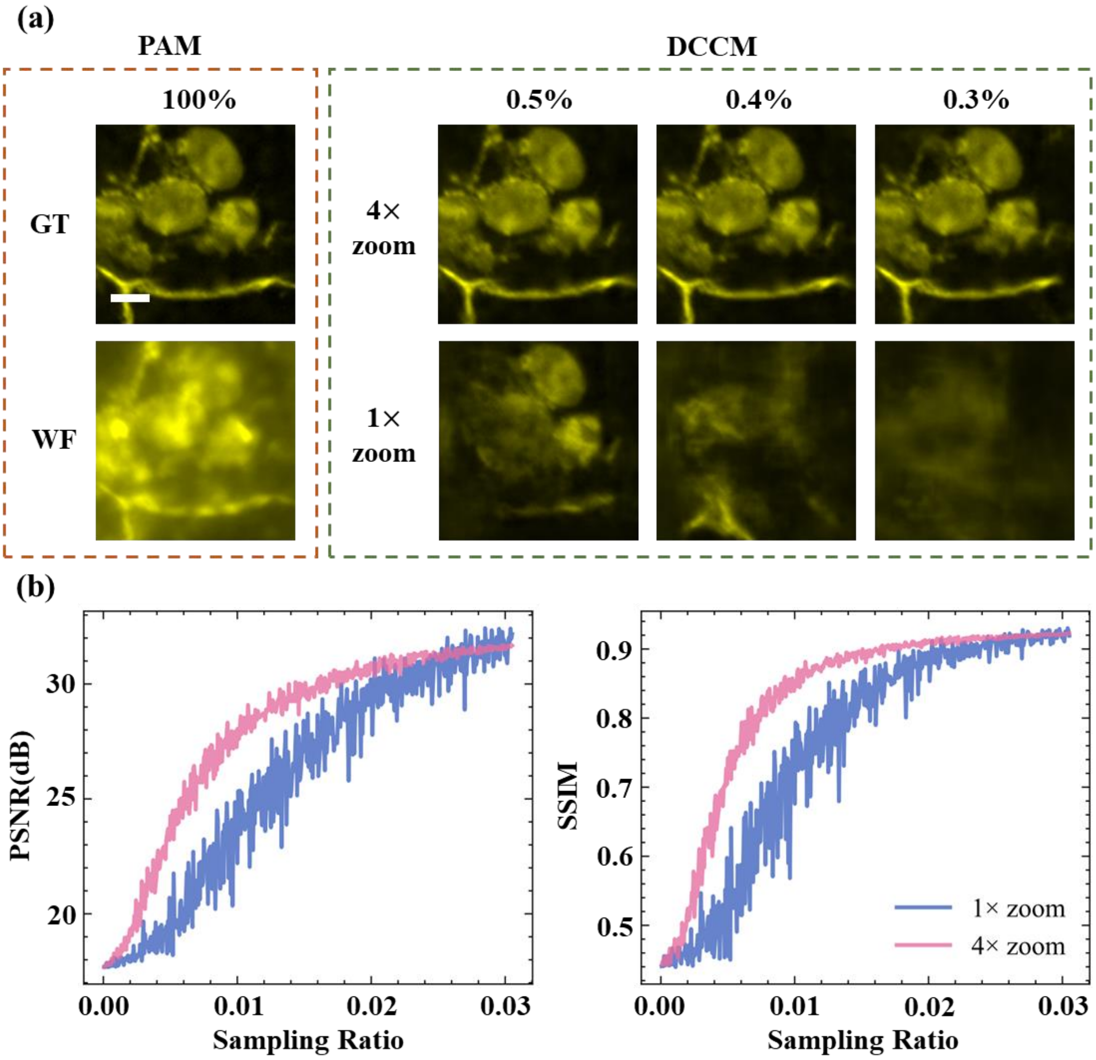


**Figure S8**. **The high-speed zoom imaging of potato tuber autofluorescence.** (a) Experimental results of potato tuber autofluorescence in our 4× and 1× zoom imaging modes with different sampling ratios of 0.5%, 0.4%, and 0.3%. (b) The curves of peak signal-to-noise ratio (PSNR) and structural similarity index measure (SSIM) scores with different sampling ratios. The corresponding scale bar is 20 $\mu m$. PAM: programmable array microscopy; DCCM: deep compressive confocal microscope; GT: ground truth; WF: wide field.

**Supplementary Note 9.**

**The perspective transformation algorithm**

The relative position between the DMD and the sCMOS are shown in Fig. S9(a). That is, the DMD pattern mapping to the sCMOS can be seen as a three-dimensional projective transformation as shown in Fig. S9(b). Therefore, the mapping relation of points from the DMD plane to the sCMOS plane can be expressed by a homographic matrix as follows:

$$\begin{bmatrix} \alpha \\ \beta \\ \theta \end{bmatrix} = \begin{bmatrix} r_{11} & r_{12} & r_{13} \\ r_{21} & r_{22} & r_{23} \\ r_{31} & r_{32} & r_{33} \end{bmatrix} \cdot \begin{bmatrix} x \\ y \\ 1 \end{bmatrix}, \quad (6)$$

where the $[\alpha \quad \beta \quad \theta]^{\mathrm{T}}$ and $[x \quad y \quad 1]^{\mathrm{T}}$ are the homogeneous coordinates of the DMD plane and sCMOS plane, $r_{ij}$ is the $i$th row and the $j$th column of the projective mapping homographic matrix. Here, we use a telecentric lens to increase the available depth of field which turns the tilted DMD plane into focus ($\theta \approx 1$). This could also be realized by tilting the sCMOS according to the Scheimpflug condition [6]. Therefore, when four points (A, B, C and D) on the DMD plane and the corresponding points (a, b, c, and d) on the sCMOS plane are known, the homographic matrix can be calculated by the perspective projections algorithm [7]. After the homographic matrix is obtained, the arbitrary point in the DMD plane can be calculated according to its corresponding point in the sCMOS plane, as shown in Fig. S9(c).

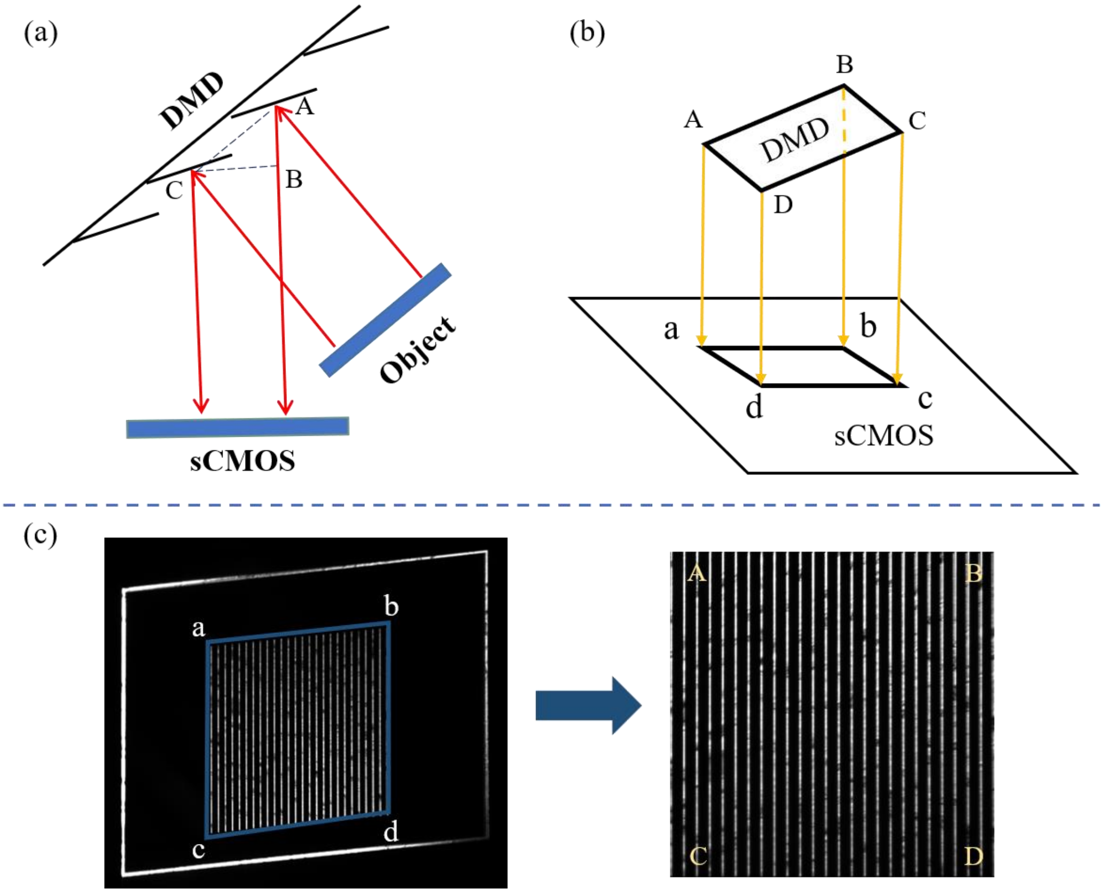


**Figure S9. Schematic diagram of the projection transformation principle.** (a) The schematic of the relative position between the digital micromirror device (DMD) and scientific complementary metal-oxide-semiconductor (sCMOS). (b) The schematic diagram of the projection transformation principle. (c) The result of pixel alignment.

**Supplementary Note 10.**

**Confocal imaging schemes**

In this study, we adopt the principle of PAM imaging to acquire the confocal image utilized as the ground truth (GT) for network training. The DMD is placed in the image plane of the objective lens and is used as a programmable pinhole. The schematic diagram of confocal imaging principle is shown in Fig. S10(a). The in-focus fluorescence passes through the objective lens and reaches the digital micro-reflector, being the $+12°$ direction state, and then is reflected to the sCMOS. However, since the out-focus fluorescence reaches the digital micro-reflector in the $-12°$ direction state, it cannot be reflected to sCMOS. An important advantage of the confocal fluorescence microscopy utilizing this DMD is the flexibility of the illumination pattern. In this work, we use the multi-point or multi-line pattern as the pinhole pattern, as shown in Fig. S10(b). In addition, we provide a graphical representation of the relationship between the pinhole separation distance and the axial resolution. The plot in Fig. S10(c) shows the total intensity for recorded image as a function of axial scan position. It clearly shows that the z-axis resolution is highest when the two pinholes are separated by 16 micromirror elements. The z-axis resolution gradually becomes smaller as the separation of pinhole decreases. The size of each micromirror element is $13.68\mu m \times 13.68\mu m$.

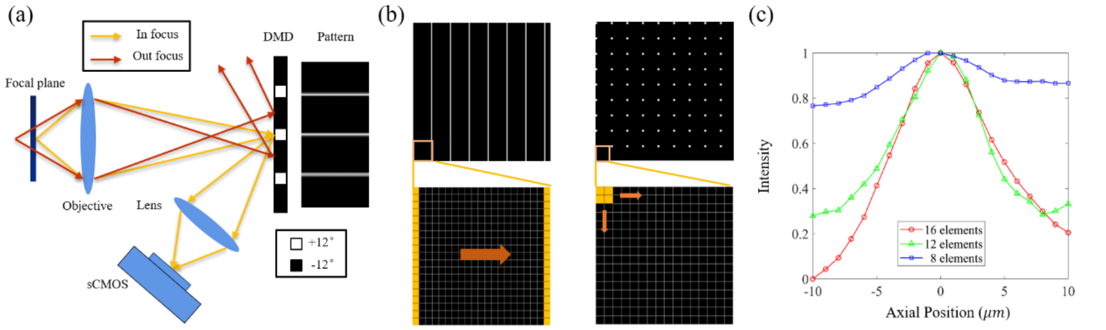


**Figure S10**. **Implementation and analysis of confocal imaging.** (a) The schematic of confocal imaging. (b) The pinhole scanning pattern. (c) Plots of axial point spread function of the system corresponding to different separation of the pinholes. DMD: digital micromirror device; sCMOS: scientific complementary metal-oxide-semiconductor.

# Supplementary References


1. Shi W, Caballero J, Huszár F, Totz J, Aitken AP, Bishop R, Rueckert D, Wang Z. Real-time single image and video super-resolution using an efficient sub-pixel convolutional neural network. In Proceedings of the IEEE conference on computer vision and pattern recognition, pp. 1874-1883. (2016).
2. Zhang J., Chen B., Xiong R., Zhang Y. Physics-Inspired Compressive Sensing: Beyond deep unrolling. *IEEE Signal Proc. Mag.* **40**, 58-72 (2023).
3. Song J., Chen B., Zhang J. Memory-Augmented Deep Unfolding Network for Compressive Sensing. *Proceedings of the 29th ACM International Conference on Multimedia* (2021).
4. Ba J., Kiros J. R., Hinton G. E. Layer Normalization. *ArXiv* **abs/1607.06450** (2016).
5. Chen L, Chu X, Zhang X, Sun J. Simple baselines for image restoration. In European conference on computer vision, pp. 17-33. Cham: Springer Nature Switzerland (2022).
6. Feng W., Zhang F., Wang W., Xing W., Qu X. Digital micromirror device camera with per-pixel coded exposure for high dynamic range imaging. *Appl. Opt.* **56**, 3831-3840 (2017).
7. Anderson JA. Canonical description of the perspective projections. In Vision Geometry, vol. 1832, pp. 300-311. SPIE (1993).
