# Supplementary material for: Compressive confocal microscopy imaging at the single-photon level with ultra-low sampling ratios
Source: Commun Eng. 2024 Jun 25;3:88. doi: 10.1038/s44172-024-00236-x (PMC11199654; doi:10.1038/s44172-024-00236-x)
Supplement: Supplementary file 2 — Reporting Summary [file 44172_2024_236_MOESM2_ESM.pdf]

nature portfolio

Corresponding author(s): Yongbing Zhang

Last updated by author(s): Apr 29, 2024

Reporting Summary

Nature Portfolio wishes to improve the reproducibility of the work that we publish. This form provides structure for consistency and transparency in reporting. For further information on Nature Portfolio policies, see our Editorial Policies and the Editorial Policy Checklist.

Statistics

For all statistical analyses, confirm that the following items are present in the figure legend, table legend, main text, or Methods section.

n/a	Confirmed	
☐	☒	The exact sample size (*n*) for each experimental group/condition, given as a discrete number and unit of measurement
☐	☒	A statement on whether measurements were taken from distinct samples or whether the same sample was measured repeatedly
☐	☒	The statistical test(s) used AND whether they are one- or two-sided<br>*Only common tests should be described solely by name; describe more complex techniques in the Methods section.*
☒	☐	A description of all covariates tested
☒	☐	A description of any assumptions or corrections, such as tests of normality and adjustment for multiple comparisons
☐	☒	A full description of the statistical parameters including central tendency (e.g. means) or other basic estimates (e.g. regression coefficient) AND variation (e.g. standard deviation) or associated estimates of uncertainty (e.g. confidence intervals)
☒	☐	For null hypothesis testing, the test statistic (e.g. *F*, *t*, *r*) with confidence intervals, effect sizes, degrees of freedom and *P* value noted<br>*Give P values as exact values whenever suitable.*
☒	☐	For Bayesian analysis, information on the choice of priors and Markov chain Monte Carlo settings
☐	☒	For hierarchical and complex designs, identification of the appropriate level for tests and full reporting of outcomes
☐	☒	Estimates of effect sizes (e.g. Cohen's *d*, Pearson's *r*), indicating how they were calculated

Our web collection on statistics for biologists contains articles on many of the points above.

Software and code

Policy information about availability of computer code

Data collection	HCImageLive was used for data collection with sCMOS.
Data analysis	All data analyses were performed with customized MATLAB (MathWorks, MATLAB 2018) programs. The implementation of the DCCM-Net is based on Python 3.9. All the codes are available upon request.

For manuscripts utilizing custom algorithms or software that are central to the research but not yet described in published literature, software must be made available to editors and reviewers. We strongly encourage code deposition in a community repository (e.g. GitHub). See the Nature Portfolio guidelines for submitting code & software for further information.

Data

Policy information about availability of data

All manuscripts must include a data availability statement. This statement should provide the following information, where applicable:

- Accession codes, unique identifiers, or web links for publicly available datasets
- A description of any restrictions on data availability
- For clinical datasets or third party data, please ensure that the statement adheres to our policy

Data underlying the results presented in this paper are not publicly available but may be obtained from the authors upon reasonable request.

Human research participants

Policy information about [studies involving human research participants and Sex and Gender in Research.](#)

Reporting on sex and gender	Not relevant, as there were no such experimental groups in this study.
Population characteristics	Not relevant, as there were no such experimental groups in this study.
Recruitment	Not relevant, as there were no such experimental groups in this study.
Ethics oversight	Not relevant, as there were no such experimental groups in this study.

Note that full information on the approval of the study protocol must also be provided in the manuscript.

Field-specific reporting

Please select the one below that is the best fit for your research. If you are not sure, read the appropriate sections before making your selection.

☒ Life sciences ☐ Behavioural & social sciences ☐ Ecological, evolutionary & environmental sciences

For a reference copy of the document with all sections, see nature.com/documents/nr-reporting-summary-flat.pdf

Life sciences study design

All studies must disclose on these points even when the disclosure is negative.

Sample size	For the experiment of sample, We collect 1,200 128×128 patches for training, and about 50 128×128 patches for testing.
Data exclusions	No data were excluded from analyses
Replication	Our experiments were tested over four types of biological samples, showing similar performance.
Randomization	Not relevant, as there were no such experimental groups in this study.
Blinding	Not relevant, as there were no such experimental groups in this study.

Reporting for specific materials, systems and methods

We require information from authors about some types of materials, experimental systems and methods used in many studies. Here, indicate whether each material, system or method listed is relevant to your study. If you are not sure if a list item applies to your research, read the appropriate section before selecting a response.

Materials & experimental systems

n/a	Involved in the study	
☒	☐	Antibodies
☐	☒	Eukaryotic cell lines
☒	☐	Palaeontology and archaeology
☒	☐	Animals and other organisms
☒	☐	Clinical data
☒	☐	Dual use research of concern

Methods

n/a	Involved in the study	
☒	☐	ChIP-seq
☒	☐	Flow cytometry
☒	☐	MRI-based neuroimaging

Eukaryotic cell lines

Policy information about [cell lines and Sex and Gender in Research](#)

Cell line source(s)	NIH3T3 cells and MC38 cells
Authentication	These cell lines are not authentisized.
Mycoplasma contamination	These cell lines are not tested for mycoplasma contenmination.

Commonly misidentified lines<br>(See ICLAC register)	NIH3T3 cells and MC38 cells are not listed in the version 11 (released 8 June 2021) "Misidentified cell lines where no authentic stock is known" table from ICLAC database.
